# Inter-observer reproducibility of classical lobular neoplasia (B3 lesions) in preoperative breast biopsies: a study of the Swiss Working Group of breast and gynecopathologists

**DOI:** 10.1007/s00432-020-03195-w

**Published:** 2020-03-30

**Authors:** Linda Moskovszky, Barbara Berger, Achim Fleischmann, Thomas Friedrich, Birgit Helmchen, Meike Körner, Tilman T. Rau, Zsuzsanna Varga

**Affiliations:** 1 Institute of Pathology and Molecular Pathology, University Hospital Zurich, Schmelzbergstrasse 12, 8091 Zurich, Switzerland; 2 Pathologie Länggasse, Ittigen, Switzerland; 3 Pathology Institute, Cantonal Hospital Thurgau, Münsterlingen, Switzerland; 4 Pathology Institute Regebogen, Kreuzligen, Switzerland; 5 Pathology Institute, Triemlispital, Zurich, Switzerland; 6 Pathology Institute, Cantonal Hospital Aarau, Aarau, Switzerland; 7 Institute of Pathology, University Hospital Bern, Bern, Switzerland

**Keywords:** Lobular neoplasia, Atypical lobular hyperplasia, Lobular carcinoma in situ, B3 lesion, Inter-observer variability

## Abstract

**Purpose:**

Classical type of lobular neoplasia (LN) spans a spectrum of disease, including atypical lobular hyperplasia (ALH) and lobular carcinoma in situ (LCIS), classical lobular neoplasia (LN), and the three-tiered classification of lobular intraepithelial neoplasia (LIN-1, -2, -3). This study addressed inter-observer variability of classical lobular neoplasias (LN) (B3 lesions) in preoperative breast biopsies among breast and gynecopathologists

**Methods:**

A retrospective, observational, cross-sectional study was conducted. 40 preoperative digital images of breast core/vacuum biopsies were analyzed by eight experienced breast- and gynecopathologists. Evaluation criteria were ALH, LCIS, LN classic, LIN-1, LIN-2, LIN-3, focal B3 (one focus), extensive B3 (> one focus). Kappa-index and Chi-square tests were used for statistics. Digital scanned slides were provided to each participant. Agreement between the categories was defined as at least six of eight (cut-off 75%) concordant diagnoses.

**Results:**

The highest agreement between eight pathologists was reached using the category lobular neoplasia (LN, classical), 26/40 (65%) cases were diagnosed as such. Agreements in other categories was low or poor: 12/40 (30%) (ALH), 9/40 (22%) (LCIS), 8/40 (20%) (LIN-1), 8/40 (20%) (focal B3), 3/40 (7.5%) (LIN-2), and 2/40 (5%) (extensive B3). Chi-square-test (classical LN versus the other nomenclatures) was significant (*p* = 0.001137).

**Conclusion:**

Our data suggest that among Swiss breast pathologists, the most reproducible diagnosis for B3 lobular lesions is the category of classical LN. These data further support lack of consistent data in retrospective studies using different terminologies. Validation of reproducible nomenclature is warranted in further studies. This information is useful especially in view of retro- and prospective data analysis with different diagnostic categories.

## Introduction

Lobular neoplasia of the breast comprises a large variation in atypical epithelial proliferation within the acinar breast structures (Foote and Stewart 1941; Haagensen et al. 1978; King et al. 2015; King and Reis-Filho 2014; Lakhani et al. 2016; Tavassoli 2003; WHO 2019; Wen and Brogi 2018). In low-grade lesions, several existing alternative terminologies such as classical lobular neoplasia (LN classical type) including both atypical lobular hyperplasia (ALH) and lobular carcinoma in situ (classical LCIS) and the so-called Lobular Intraepithelial Neoplasia (LIN) covering LIN-1, LIN-2, LIN-3 (Lakhani et al. 2016; Tavassoli 2003; WHO 2019; Rageth et al. 2016, 2019) allow to classify the same lobular breast lesion with different diagnostic terms. Although clinical management of these alternative low-grade terminologies is quite similar and all are considered as risk factors and non-obligate precursor for breast cancer, there is still no single pathological factor to predict upgrade, progression, and/or local recurrence (Foote and Stewart 1941; Haagensen et al. 1978; King et al. 2015; King and Reis-Filho 2014; Lakhani et al. 2016; Tavassoli 2003; WHO 2019; Wen and Brogi 2018; Rageth et al. 2016, 2019; AGO 2019). On the contrary, high-grade lobular in situ lesions such as pleomorphic or florid LCIS/LN exhibit a biologically similar behavior and require the same management as their ductal carcinoma in situ (DCIS) counterpart (WHO 2019; Wen and Brogi 2018; AGO 2019; Shamir et al. 2019).

Inter-observer agreement data on different terminologies are sparse and these data point to improved agreement when favoring one category to more than one descriptive subgroup (AGO 2019; Choi et al. 2008; Fitzgibbons 2000; Gomes et al. 2014; Singh et al. 2018). In our study, we addressed the question on inter-observer agreement on six existing non-pleomorphic LN terminologies using 40 diagnostic LN breast core and vacuum biopsy cases with eight participating pathologists specialized in breast pathology.

## Materials and methods

40 cases of breast core- and vacuum biopsies with the diagnosis B3 lesion and lobular neoplasia were retrieved from the Institute of Pathology and Molecular Pathology, University Hospital Zurich Switzerland, in the years 2012–2013. All cases were diagnostic cases from routine histological diagnostics. The diagnosis of lobular neoplasia and B3 category was made on conventional hematoxylin–eosin (H&E) stains and confirmed with immunohistochemistry (E-Cadherin loss and/or catenin p120 cytoplasmic staining) in all cases at the time of the routine diagnostics.

The study was conducted within the project approved by the cantonal committee of the Canton Zurich (KEK-2012-554). Informed consent was not necessary as all cases were analyzed in a fully anonymized way.

### Study design

All eight participants of the study were members of the Working Group of Breast and Gynecopathology of the Swiss Society of Pathology. For the study, a digital link containing H&E images of the biopsies as well as an excel data sheet were sent to all participants. Participants were asked to assess the H&E images and to enter any of the following further diagnostic subcategory which they think would fit to the index case.

**Fig. 1:**
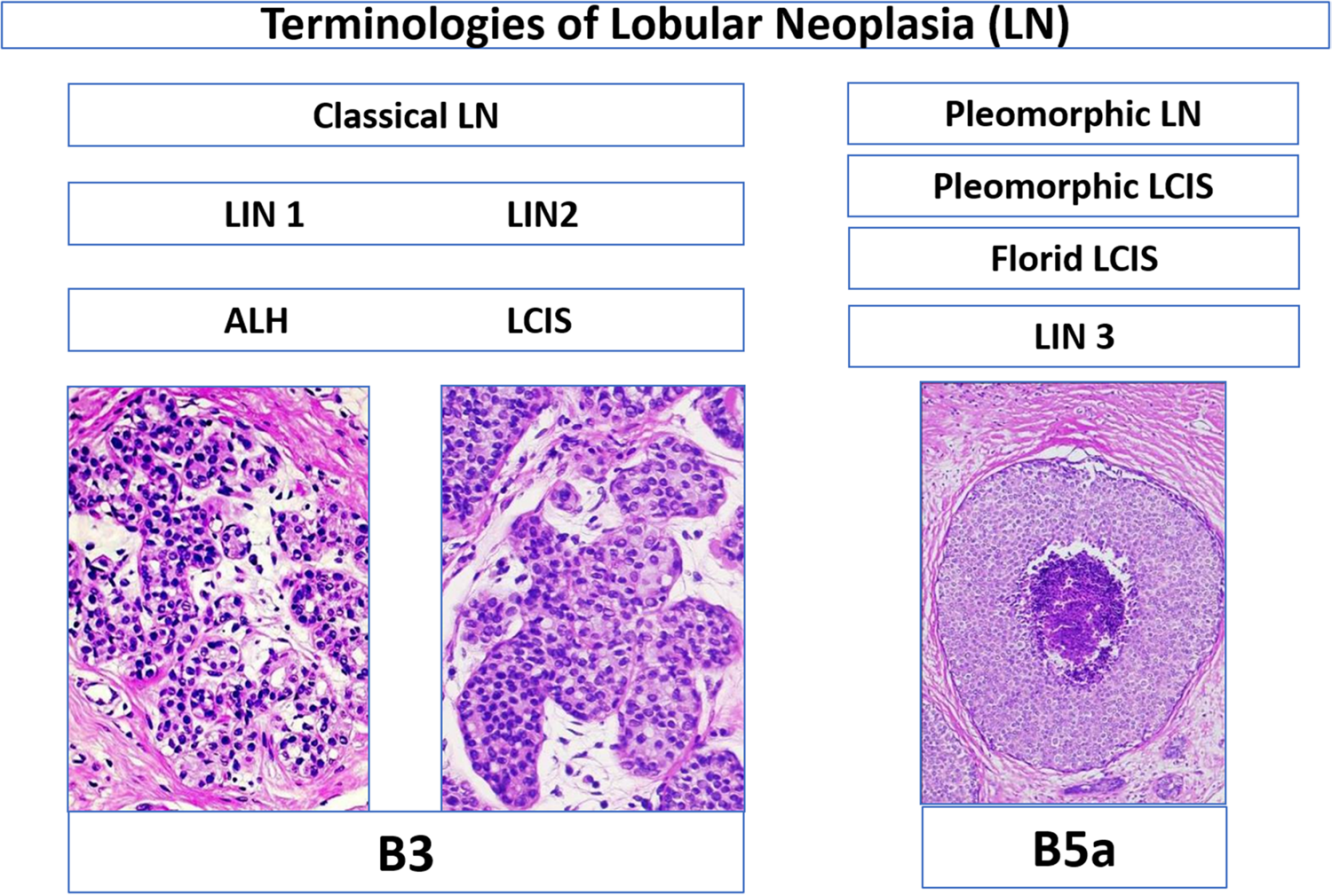
Flowchart of different terminologies of lobular neoplasia and their designation in the B classification. *LN* lobular neoplasia, *ALH* atypical lobular hyperplasia, *LCIS* lobular carcinoma in situ, *LIN* lobular intraepithelial neoplasia

**Fig. 2:**
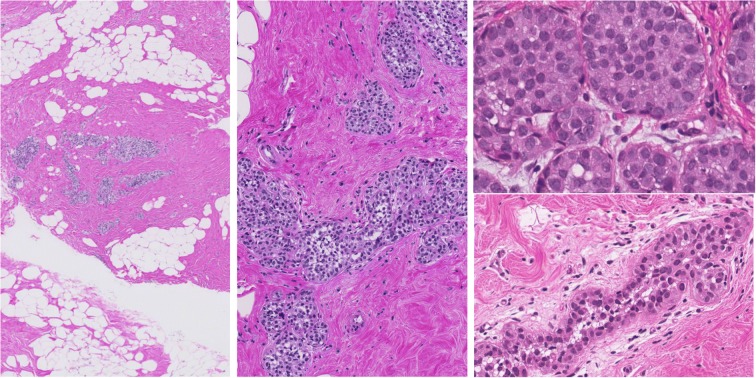
Illustration of different histological appearances of lobular neoplasia of classical type. H&E stain

These categories were named as follows: atypical lobular hyperplasia (ALH), lobular carcinoma in situ of classical type (LCIS classical type), lobular intraepithelial neoplasia I, II, III (LIN-I, LIN-II, LIN-III), lobular neoplasia of classical type (LN, classical type), focal or extensive classical LN (one or more than one focus of LN), and others (different from the mentioned category). A given case could be classified in multiple categories by the participant (Figs. 1 and 2).

An agreement in classification of a lesion was defined if a category was entered by at least six out of participating eight pathologists.

### Statistical analyses

Results were analyzed using the Chi-square statistics and Kappa Fleiss to compare agreed diagnostic categories.

## Results

**Fig. 3:**
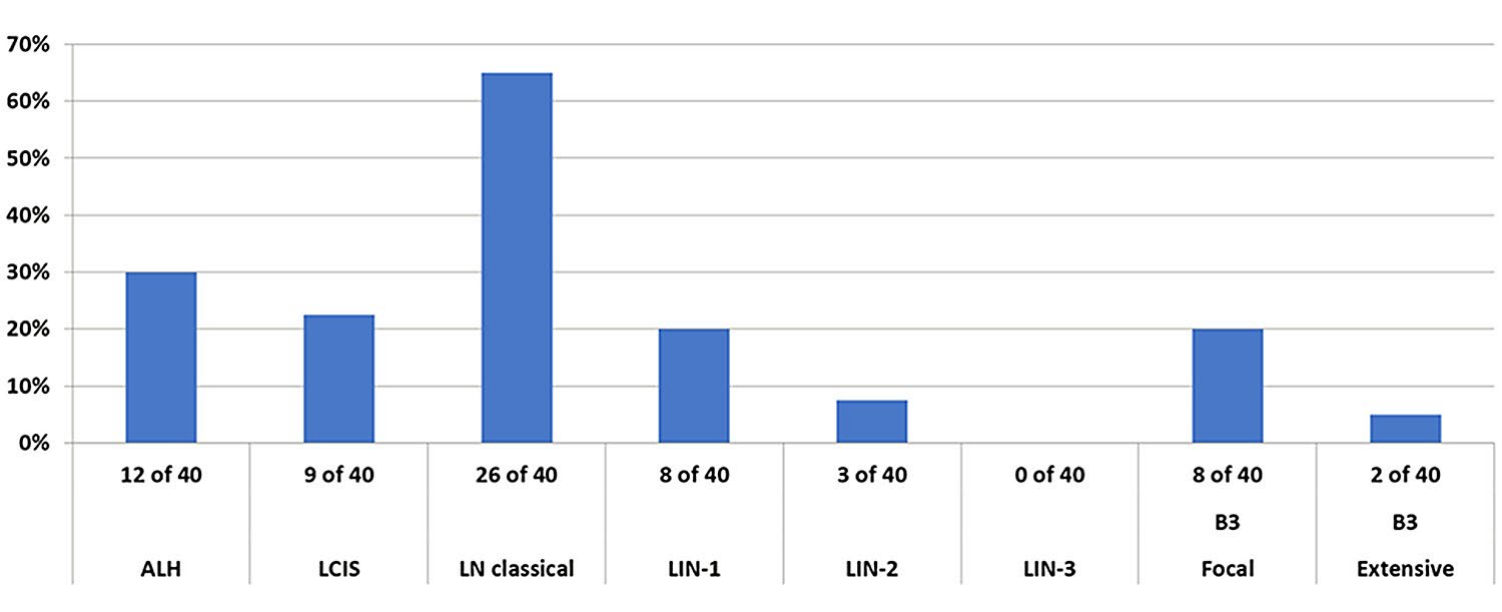
Distribution on agreed terminological categories on 40 diagnostic cases with lobular neoplasia. The highest agreement was reached at classical lobular neoplasia (*p* = 0.001137)

The highest agreement between eight pathologists was reached using the category lobular neoplasia (LN, classical), 26/40 (65%) cases were diagnosed as such. Agreements in other categories was low or poor: 12/40 (30%) (ALH), 9/40 (22%) (LCIS), 8/40 (20%) (LIN-1), 8/40 (20%) (focal B3), 3/40 (7.5%) (LIN-2), and 2/40 (5%) (extensive B3). Chi-square statistic was significant for the differences on agreement between classical LN versus the other nomenclatures (*p* = 0.001137). Kappa Fleiss could not be applied due to multiple answers per case (Fig. 3).

## Discussion

This is the first study to address reproducibility among existing established designations for classical B3 lobular lesions. In this study, we could show that different existing terminological categories all describing classical type lobular neoplasia of the breast are unequally reproduced among expert breast pathologists. Among the known diagnostic categories such as atypical lobular hyperplasia, lobular carcinoma in situ of classical type, lobular intraepithelial neoplasia I, II, III, and lobular neoplasia of classical type, the diagnostic agreement varies from 5–65%. The terminology ‘lobular neoplasia of classical type’ reached the highest agreement with 65% among breast pathologists.

The term lobular neoplasia encompasses a spectrum of histological lesions with differences in extent and the degree of nuclear atypia (Foote and Stewart 1941; Haagensen et al. 1978; King et al. 2015; King and Reis-Filho 2014; Lakhani et al. 2016; Tavassoli 2003; WHO 2019; Wen and Brogi 2018; Jorns et al. 2014). The original paper by Foote and Stewart from 1941 described and defined the morphological criteria and differences between ALH and LCIS as both lesions exhibiting the same low-grade monotonous nuclear atypia but differing quantitatively in their acinar involvement (Foote and Stewart 1941; King et al. 2015; King and Reis-Filho 2014; Wen and Brogi 2018; Jorns et al. 2014). Alternative terminologies such as classical lobular neoplasia (LN classical type) including both ALH and LCIS as well as the term Lobular Intraepithelial Neoplasia (LIN) covering LIN-1, LIN-2, LIN-3 as consecutive morphological categories represent a further approach to classify the same lobular breast lesions (Lakhani et al. 2016; Tavassoli 2003; WHO 2019; Rageth et al. 2016; AGO 2019). These lesions are considered both as risk-factor and non-obligate precursor for breast cancer in terms of uncertain malignant potential also categorized as B3 lesions in some guidelines (Lakhani et al. 2016; Tavassoli 2003; WHO 2019; Wen and Brogi 2018; Rageth et al. 2016, 2019). Long-term cumulative risk of classical LN for breast cancer is 1–2% per year, resulting in 8–10 × relative risk for LCIS and 4–5 × relative risk for ALH (King et al. 2015; King and Reis-Filho 2014; Lakhani et al. 2016; WHO 2019; Rageth et al. 2016).

Morphological variants with high nuclear grade, with the presence of necrosis or with extensive involvement of the multiple acini, are considered as separate entities and are designated as pleomorphic LCIS/LN, florid LCIS/LN, or LIN-3, and are also categorized as B5a category (non-invasive pre-malignant lesion) (Lakhani et al. 2016; Tavassoli 2003; WHO 2019; Rageth et al. 2016; 2019). Pleomorphic LCIS/LN, florid LCIS/LN, or LIN-3 can morphologically mimic solid type of DCIS, but represent molecularly distinct entities (WHO 2019; Wen and Brogi 2018; Shamir et al. 2019). However, lobular lesions in the B5a category behave biologically similar as their DCIS counterpart, have higher risk for local recurrence and progression to invasive cancer, are more often Her2 positive, and therefore, their clinical management is very similar to DCIS (WHO 2019; Wen and Brogi 2018; Shamir et al. 2019).

On the contrary, classical lobular neoplasia forms are known to have a different biological behavior in terms of local recurrence and development of synchronous or subsequent breast cancer than the high-grade variants (King et al. 2015; King and Reis-Filho 2014; Wen and Brogi 2018; Rageth et al. 2016, 2019; Schmidt et al. 2018). Upgrade rate to in situ or invasive cancer in open excision specimens has been conflictingly reported in the literature varying from 0 to 25% in some papers up to 50% (Rageth et al. 2016, 2019; Schmidt et al. 2018). No association with common clinical risk factors as positive family history or age can be linked to clinical behavior, and until now, no single histopathological factor could predict upgrade or development of concurrent or subsequent breast cancer (King et al. 2015; Rageth et al. 2016, 2019). However, clinical management of classical lobular neoplasia has undergone relevant modifications during the last decade, including the identification of imaging target lesions as visible lesions and the histological association to mammographic calcifications into the management workflow (Rageth et al. 2016, 2019; AGO 2019). Current therapeutic guidelines recommend open excision for classical LN forms in breast core biopsies if there is a target lesion on imaging and in case of any inconsistency between imaging modalities and pathological assessment (Rageth et al. 2016, 2019; AGO 2019). In all other classical LN cases, a conservation approach with a high-risk senological follow-up is acceptable, especially in diagnoses made by breast vacuum biopsy and if the radiological target has been removed (Rageth et al. 2016, 2019; AGO 2019).

Although this therapeutic approach has been the standard for all classical LN forms, until now, guidelines do not consider different subgroups of classical lobular neoplasia as ALH vs LCIS or LIN-1 vs LIN-2 (Rageth et al. 2016, 2019; AGO 2019). Classical forms of B3 LN lesions are mainly subjected to a very similar therapeutic workflow (Rageth et al. 2016, 2019; AGO 2019). The AGO (2019) guidelines specifically do not recommend the distinction between LIN-1 and LIN-2, because prognostic differences have not adequately been documented and proven until now, even though absolute risk for breast cancer development differs between ALH and classical LCIS (AGO 2019).

Reproducibility issues concerning a wide spectrum of pre-malignant breast lesions, biomarkers, or degree of atypia have been the subject of several previous papers (Shamir et al. 2019; Choi et al. 2008; Fitzgibbons 2000; Gomes et al. 2014; Allison et al. 2016; Carney et al. 2016; Elmore et al. 2015, 2016a, b; O'Malley et al. 2006; Onega et al. 2017; Schuh et al. 2010; Sloane et al. 1998; Tan et al. 2005; Wells et al. 2000). The use of immunohistochemistry with aberrant E-Cadherin staining combined with morphological criteria led to an excellent agreement (86.9%) of correctly classifying in situ or invasive lobular carcinomas and rule out morphological differential diagnoses of duct lesions such as solid-type DCIS or invasive ductal carcinomas (Choi et al. 2008). Gomes et al. (2014) reported differential inter-observer variability among pre-malignant breast lesions including atypical ductal hyperplasia, columnar cell lesions, lobular neoplasia, and DCIS in a large series of second opinions. In this paper, ALH and LCIS had both had a substantial inter-observer agreement after external review (Kappa 0.62 vs 0.66) (Gomes et al. 2014). Similar data were observed in a study by Fitzgibbons, where ALH and LCIS were inadequately classified when considered as separate entities (17% and 58% correct diagnoses); however, diagnostic accuracy improved to 74% when both lesions were categorized as one entity (Fitzgibbons 2000). Our results corroborate with these observations, single entities such as ALH, classical LCIS, or LIN-1 or LIN-2 did not result in satisfactory agreement (10–30% agreement), only using one category as classical lobular neoplasia including all B3 entities had an improved agreement (65%), which was also statistically significant. Singh et al. (2018) reported on a similar trend on improved reproducibility when pleomorphic and florid lobular carcinoma in situ were grouped into one diagnostic category.

As was also suggested by Haagensen et al. (1978) more than 4 decades ago and also supported by the current study, insufficient reproducibility between slightly different histological entities can be improved using one category as classical LN.

Similar issues were addressed in DCIS in several previous studies (Onega et al. 2017; Schuh et al. 2010; Sloane et al. 1998; Wells et al. 2000). Comparing three DCIS classification systems, the van Nuys system resulted in the highest diagnostic agreement in the Sloane project and by Shuh et al. (Kappa 0.42 and 0.37), although the final histological grading of DCIS was better reproducible using the Holland classification in the other studies (Kappa 0.53) (Schuh et al. 2010; Sloane et al. 1998; Wells et al. 2000). Applying a two-tiered grading system in DCIS (as low vs high grade) as opposed with reference diagnoses, high-grade DCIS proved to be more robust than low grade (83% vs 46% agreement with reference diagnoses) (Onega et al. 2017).

Reproducibility issues in atypical ductal breast lesions, such as columnar cell lesions, flat epithelial atypia (FEA), atypical ductal hyperplasia (ADH), or DCIS show a similarly unequal trend (Allison et al. 2016; Carney et al. 2016; O'Malley et al. 2006; Tan et al. 2005). Agreement for FEA varies in the literature from poor (Kappa 0.27) to excellent (Kappa 0.83) (O'Malley et al. 2006; Tan et al. 2005). Regarding ADH, solid or micropapillary pattern with borderline cytological atypia was shown to be associated with lower agreement than those with cribriform pattern and clearly monotonous atypia (Allison et al. 2016).

Differences and agreements in pathologist’s opinions in a broader range of breast surgical specimens were documented in several earlier papers (Carney et al. 2016; Elmore et al. 2015; 2016a; b). Under- and overestimation of atypia and consistency in overall agreement with diagnostic standards were found between non-academic and academic pathologists (77.6% vs. 46%) (Carney et al. 2016). Elmore et al. reported misinterpretation in terms of atypia as highest after one single evaluation (52.2%) and the level of diagnostic concordance as highest in invasive carcinoma and lowest for DCIS and atypia (Elmore et al. 2015, 2016a, b).

In summary, our results show that existing different terminologies on classical form of LN in general have a poor-to-substantial agreement among expert breast pathologists on the same lesion, except when using a single category of classical lobular neoplasia. Regarding therapeutic approaches, until now, there is no difference in management between ALH, LCIS, LIN-1, and LIN-2 or classical LN (Rageth et al. 2016, 2019; AGO 2019). Decisions for open surgery currently require discordant lesions between histology and imaging, a suspicious mass lesion in imaging or inadequately removed target lesions by vacuum-assisted biopsies (Rageth et al. 2016, 2019; AGO 2019). Although until now no single histopathological factor of classical LN diagnosis could be identified to predict upgrade or local recurrence, helpful morphological ancillary tools such as information on associated calcifications in LN and a rough LN extension in breast core and vacuum-assisted biopsies can contribute to management decisions and possibly enable image-based senological follow-up in larger subset of LN cases.
